# *Pseudomonas aeruginosa* PA5oct Jumbo Phage Impacts Planktonic and Biofilm Population and Reduces Its Host Virulence

**DOI:** 10.3390/v11121089

**Published:** 2019-11-23

**Authors:** Tomasz Olszak, Katarzyna Danis-Wlodarczyk, Michal Arabski, Grzegorz Gula, Barbara Maciejewska, Slawomir Wasik, Cédric Lood, Gerard Higgins, Brian J. Harvey, Rob Lavigne, Zuzanna Drulis-Kawa

**Affiliations:** 1 Department of Pathogen Biology and Immunology, Institute of Genetics and Microbiology, University of Wroclaw, 51-148 Wroclaw, Poland; tomasz.olszak@uwr.edu.pl (T.O.); danis-wlodarczyk.1@osu.edu (K.D.-W.); grzegorz.gula@uwr.edu.pl (G.G.); barbara.maciejewska@uwr.edu.pl (B.M.); 2 Laboratory of Gene Technology, KU Leuven, 3001 Heverlee, Belgium; cedric.lood@kuleuven.be (C.L.); rob.lavigne@kuleuven.be (R.L.); 3 Department of Biochemistry and Genetics, Institute of Biology, The Jan Kochanowski University in Kielce, 25-406 Kielce, Poland; arabski@ujk.kielce.pl; 4 Department of Molecular Physics, Institute of Physics, The Jan Kochanowski University in Kielce, 25-406 Kielce, Poland; s.wasik@ujk.kielce.pl; 5 Laboratory of Computational Systems Biology, KU Leuven, 3000 Leuven, Belgium; 6 National Children Research Centre, Our Lady’s Children’s Hospital, Crumlin, 12 Dublin, Ireland; gerardhiggins@rcsi.ie; 7 Department of Molecular Medicine, Royal College of Surgeons in Ireland, Education and Research Centre, Beaumont Hospital, 9 Dublin, Ireland; brianharvey@rcsi.ie

**Keywords:** giant bacteriophage, *Pseudomonas aeruginosa*, biofilm, Airway Surface Liquid Infection model, phage-resistant mutants

## Abstract

The emergence of phage-resistant mutants is a key aspect of lytic phages-bacteria interaction and the main driver for the co-evolution between both organisms. Here, we analyze the impact of PA5oct jumbo phage treatment on planktonic/cell line associated and sessile *P. aeruginosa* population. Besides its broad-spectrum activity and efficient bacteria reduction in both airway surface liquid (ASL) model, and biofilm matrix degradation, PA5oct appears to persist in most of phage-resistant clones. Indeed, a high percentage of resistance (20/30 clones) to PA5oct is accompanied by the presence of phage DNA within bacterial culture. Moreover, the maintenance of this phage in the bacterial population correlates with reduced *P. aeruginosa* virulence, coupled with a sensitization to innate immune mechanisms, and a significantly reduced growth rate. We observed rather unusual consequences of PA5oct infection causing an increased inflammatory response of monocytes to *P. aeruginosa*. This phenomenon, combined with the loss or modification of the phage receptor, makes most of the phage-resistant clones significantly less pathogenic in in vivo model. These findings provide new insights into the general knowledge of giant phages biology and the impact of their application in phage therapy.

## 1. Introduction

The dynamic interactions between bacteriophages and their hosts are often compared to an arms race because the constant selection pressure driving the evolution of both groups. There are many mechanisms that protect bacteria from phage infection, both at the cellular and population level [1]. Receptor modifications, resulting most often from point mutations or changes in the expression level of surface structures, lead to the inhibition of phage adsorption to bacterial cells. In the case of bacteria already infected with lysogenic phage, superinfection exclusion systems (Sie) prevent the injection of DNA of closely related viruses or phage progeny. Even if the injection of viral DNA is successful, bacteria have a number of mechanisms of foreign DNA destruction: R-M (restriction-modification), DISARM (defense island system associated with restriction modification), pAgos (prokaryote argonaute proteins) and CRISPR/Cas (clustered, regularly interspaced, short palindromic repeat). The next stage of the phage development cycle is DNA replication, which can also be halted. The BREX system (bacteriophage exclusion) is responsible for this. Finally, among bacteria there is also the Abi system (abortive infection), in which the development of the phage is inhibited by the programmed death of a bacterial cell and it protects the entire population. In addition to specialized phage defense mechanisms, bacteria also use physical shields. Commonly produced by Gram-negative bacteria OMVs (outer-membrane vesicles) mislead attacking viruses that inject their genetic material into an empty vesicle [2]. The bacterial biofilm, which mechanically limits the access to the receptors recognized by phages, also plays a very important protective role. Therefore, many phages are equipped witch virion-associated enzymes (so-called depolymerases) degrading the biofilm matrix [3].

Another aspect of phage biology research is its potential application to fight pathogenic bacteria. Finding a solution to the antibiotic resistance problem is one of the greatest challenges of modern science and medicine, and the search for alternative strategies to antibacterial therapy has led to a renewed appreciation of bacteriophages [4]. Phage therapy efficacy studies [5,6,7] and recent advances in the regulatory frame, in which phage therapy can be adopted as part of “Magistral preparations” [8] have shifted the focus from proving the efficacy of phage therapy to its operational implementation, while expanding the number of available phage isolates essential for the design of therapeutic cocktails.

Among the phages currently evaluated for therapeutic applications are the jumbo phages, defined by long dsDNA genomes in excess of 200 kb [9]. Jumbo phages are commonly found in [10] commercial phage therapy products, owing to their broad host range. The large coding potential of jumbo phage allows them to be (partly) independent from the host enzymes, empowering their expanded host range [11]. However, some Jumbo phages are marked with a high frequency of transduction, which impacts the evolution of bacteria and raises questions on the safety of their use in therapy [12]. The first jumbo phage was discovered over 40 years ago (*Bacillus* bacteriophage G), but the frequency of giant phages isolation remains rather low (less than 90 complete genomes in GenBank database) [13]. They form an incredibly diverse group, and new isolates of jumbo phages generally show low similarity to those present in public databases. They are also poorly characterized functionally, with annotated genomes that contain a vast majority of genes with undefined function [14].

The first sequenced jumbo phage genome specific for *Pseudomonas aeruginosa* was phiKZ—a giant lytic myovirus with a broad host range, isolated in Kazakhstan. Its large capsid (120 nm in diameter) encloses a linear, circularly permuted, terminally redundant genome (280,334 bp, 36.8% G+C). Capacity of the capsid combined with a large genome allows the phiKZ phage to carry large fragments of bacterial DNA, contributing to horizontal gene transfer through the process of generalised transduction [15]. This phage has become a hallmark example for structural analysis of phage particle and for genetic and structural analysis [16,17,18]. Currently, over twenty giant *Pseudomonas* bacteriophages within the diverse *Phikzvirus* genus have been isolated [12,19,20].

*P. aeruginosa* phage PA5oct was isolated from sewage samples in Wroclaw, Poland. It is a representative of completely new genus of the *Myoviridae* family. The analysis of virion morphology (TEM micrograph) and genome size ranks PA5oct phage among the largest known bacterial viruses. The head diameter of PA5oct is about 131 nm and its tail is about 136 nm long [21]. It has a linear dsDNA genome containing 286,783 bp, making it the third largest genome of *Pseudomonas* phage (Genbank MK797984), and for which a comprehensive temporal transcriptome analysis, structural proteomics analysis, and host transcription response has been studied [22].

In this study we assessed the influence of PA5oct on a population of *Pseudomonas aeruginosa*, both planktonic and sessile. We evaluated the antibacterial potential in an advanced Airway Surface Liquid infection model that mimics in vitro the normal and CF lung environments. The efficacy in biofilm eradication was determined using an established laser interferometry technique to measure the biofilm matrix degradation. We also analyzed the short- and long-term consequences of an apparent process of phage maintenance in the bacterial population, focusing on the emergence of phage-resistant clones, cross-resistance to other non-related phages and the influence of PA5oct on bacterial virulence.

## 2. Materials and Methods

### 2.1. Bacteriophages Propagation and Purification

Phage PA5oct was propagated as previously described by Danis-Wlodarczyk et al. [23]. Phage lysate was purified by 0.45 and 0.22 µm filtration and the incubation with 10% polyethylene glycol 8000 (PEG 8000)—1 M NaCl according to standard procedures [24]. Finally, the CsCl-gradient ultracentrifugation was applied [25] and phage preparation was dialyzed three times for 30 min against 250 volumes of phage buffer using Slide-A-Lyzer Dialysis Cassettes G2 (Thermo Fisher Scientific Inc, Waltham, MA, USA). The phage titre was assessed using the double-agar layer technique [26] and purified samples were stored at 4 °C in the dark.

### 2.2. Phage Host Range and Phage Receptor Analysis

The phage receptor on bacterial surface was evaluated on PAO1 knock-out mutants deficient in biosynthesis of A-band and B-band O-antigen, flagella, Type IV pili, or alginate production by a standard spot test assay using phage titer of 10^5^ pfu/mL (Table 1). The bacterial susceptibility to phage-mediated lysis in the host range experiment was determined by a standard spot test assay, applying serial dilution of phage titer (10^8^–10^3^ pfu/mL) [27]. The phage host range was evaluated on 47 CF strains from the Leuven University hospital, Leuven, Belgium collection and compared to other *Pseudomonas* phages listed in Tables S1 and S2.

### 2.3. Airway Surface Liquid Infection Model

For the Airway Surface Liquid (ASL) experiments, two cell lines (NuLi-1 and CuFi-1) were used (kindly provided by prof. Zabner, University of Iowa, Iowa City, IA, USA). The NuLi-1 cell line was isolated from human airway epithelium of normal genotype whereas the CuFi-1 line comes from bronchial epithelium of CFTR ΔF508 patient. The ASL model was prepared according to methods described by Zabner [28]. The experiment was conducted as previously described [20]. In short, both cell lines were infected with 25 μL of the *P. aeruginosa* PAO1 reference strain (6.2 × 10^7^ cfu/mL), nonCF0038 isolate from burn wound (6.5 × 10^7^ cfu/mL) and CF708 small colony variant (1.0 × 10^6^ cfu/mL) and incubated for 1.5 h at 37 °C, 5% CO_2_. Next, a 25 μL volume of the PA5oct phage lysate (6 × 10^8^ pfu/mL) was added to each millicell hanging cell culture insert, which were further incubated for 1.5 h at 37 °C, 5% CO_2_. To evaluate the phage efficacy in bacteria eradication, cells after treatment were washed with PBS and CFU counts were calculated from serially dilluted apical washes plated on LB agar (Sigma-Aldrich, St. Louis, MO, USA). There were three epithelial cells viability controls prepared: a non-treated negative control, a control 1.5 hour after PA5oct phage treatment; and a control 1.5 hour after bacteria inoculation. Both cell lines were stained with 8 μM Calcien AM (Life Technologies, Camarillo, CA, USA) and 3 μM propidium iodide (PI) (Life Technologies, NY, USA) for visualisation of live and dead cells, respectively. After staining, the filter inserts were XZ scanned with a confocal microscope (Zeiss LSM 510 Meta, Jena, Germany).

The data was analyzed using the Statistica software package (StatSoft, Tulsa, OK, USA). All values were expressed as mean ± SD and significant differences between variations (denoted *p*-values < 0.05) were assessed using the Snedecor–Fisher test using one-way ANOVA.

### 2.4. Biofilm Eradication Analysis on Nephrophane Membrane

The effectiveness of biofilm matrix degradation by PA5oct phage was evaluated by laser interferometry method [20]. PAO1 biofilm was formed on the surface of Nephrophane membrane during the incubation for 72 h at 37 °C in Trypticase-Soy broth (TSB, bioMerieux, Craponne, France). After supernatant removal, the level of membrane coverage was determined as around 93% (Figure 2A). Subsequently, the biofilm was treated for four hours at 37 °C with an intact or UV-inactivated phage suspensions (5 × 10^8^ pfu/mL). After treatment, the membrane was washed with saline and examined by interferometry technique. The degradation of biofilm was assessed as the increase of biofilm matrix permeability for low-weight molecular compounds, meaning the TSB medium itself. The quantitative measurements of TSB diffusion through biofilm structure treated with phage was obtained by laser interferometry method [20,23]. The data were analyzed using the Statistica software package (StatSoft, Tulsa, OK, USA). All values obtained at the end of 40 min measurements were expressed as mean ± SD and significant differences between variations (denoted *p*-values < 0.05) were assessed using the Snedecor–Fisher test using a one-way ANOVA.

Biofilm eradication was also examined by crystal violet (0.004%) staining for 15 min as previously described [20] and tested for pyocyanin and pyoverdin/pyochelin levels in the supernatants as described below.

### 2.5. Isolation of Phage Resistant Clones from Treated Biofilm

*P. aeruginosa* PAO1 mutants resistant to PA5oct phage were isolated from PAO1 culture infected with PA5oct phage in accordance with the following procedure. In the first step, bacterial suspension in TSB was incubated (37 °C) in 96-well peg-lid plate (Nunc, Roskilde, Denmark) for 24, 48 or 72 hours enabling bacteria to form biofilm. Afterwards, the mature biofilm molded on the surface of pegs was immersed in phage PA5oct suspension (10^6^ pfu/mL) for 24 hours. Finally, pegs were washed with PBS buffer to clear away planktonic bacteria and the biofilm population was collected using an ultrasonic bath, and then plated on TSA (bioMerieux) for 24 h at 37 °C, to isolate discrete colonies. Ten randomly selected isolates from each time point, were passaged five times on TSA to confirm the stability of genetic changes. Thirty control non-treated biofilm strains were isolated in an analogous manner. Resistance to PA5 bacteriophage was confirmed in isolated clones by spot-test phage typing using a 10^5^ pfu/mL phage suspension [27]. Moreover, the clones were also tested the same way in terms of the susceptibility to other *Pseudomonas* phages: Type IV pili-dependent (phiKZ, KTN4, LUZ19), LPS-dependent (KT28, KTN6, LUZ7) and a phage with an unverified receptor (LBL3) (Table S1). All tests were performed in triplicate.

### 2.6. Pyocyanin and Pyoverdine Production

The level of pyoverdine and pyocyanine production was analyzed on a 72 h-biofilm formed on Nephrophane or selected PAO1 mutants cultured (48 h, 37 °C) in TSB medium (bioMerieux, France) in a 24-well polystyrene plate (Sarstedt, Nümbrecht, Germany) [23]. To investigate the level of pyocyanin production the supernatant absorbance was measured at λ = 695 nm. For pyoverdine production the fluorescence was measured at λex = 392 nm and λem = 460 nm.

### 2.7. Twitching Motility Assay

Solid surface bacterial movement dependent on Type IV pili, called “twitching motility” was measured according to Turnbull & Whitchurch [29]. Each bacterial suspension was transferred to agar plate using a toothpick by perpendicular stabbing through the agar layer (up to the bottom of the plate) and incubated for 48 h at 37 °C. After incubation the agar layer was removed and the 0.01% crystal violet was used for visualization of the growth zone. The growth zone results for twitching motility are presented as the mean of ten replicates.

### 2.8. Lipopolysaccharide Structure Patterns Analysis

Examination of LPS structure pattern was done using slightly modified Marolda method of extraction [30,31] and SDS-PAGE. Overnight bacterial cultures in TSB medium (bioMerieux, France) were centrifuged and adjusted to OD_600_ = 2.0 in PBS buffer. Bacterial cells were destroyed by boiling in lysis buffer (2% SDS, 4% β-mercaptoethanol, Tris, pH 6.8) and digesting with proteinase K (60 °C, 1 h). Protein debris were eliminated by incubation (70 °C, 15 min.) with an equal volume of 90% phenol. LPS containing aqueous phase was recovered using ethyl ether to remove any residual phenol. To assess the concentration of LPS used for electrophoresis, the Purpald method of KDO (3-Deoxy-D-manno-oct-2-ulosonic acid) measurement was used [32]. Subsequently, the LPS samples were separated by Tricine SDS-PAGE method (14% polyacrylamide gel with 4 M urea, 125 V). Finally, after separation, LPS samples were stained with silver according to Tsai & Frasch [33].

### 2.9. Growth Rate Measurement

The growth rate of selected isolates was estimated using the indirect method, by measuring the optical density of a 24-hour culture at a 30 min. interval. The 18-hour Trypticase-Soy Agar (TSA) plate cultures were suspended in PBS with an optical density of 0.5 McF and used to establish TSB liquid cultures in a 24-well titration plate (starting cfu/mL = 10^6^). A plate with applied cultures was placed inside of a microplate reader (Varioscan Lux, Thermo Scientific) and incubated for 24 hours. The absorbance measurement (λ = 590 nm) was performed automatically every 30 min. after shaking for 30 s. The results of the growth rate measurements were classified in two categories: HIGH (OD_590_ after 24 h > 1.5) and LOW (OD_590_ after 24 h < 1.0).

### 2.10. TLR Stimulation Profile in THP1-XBlue™ Cell Line, NF-κB/AP-1-Reporter Monocytes

The Toll-like receptors (TLRs) stimulation of monocytes, major forms of innate immune sensors, was assessed according to manufacturer protocol using THP1-XBlue™ cell line (InvivoGen, Toulouse France). THP1-XBlue™ cells derive from the human monocytic THP-1 cell line and express an NF-κB- and AP-1-inducible secreted embryonic alkaline phosphatase (SEAP) reporter gene. Upon TLR2, TLR1/2, TLR2/6, TLR4, TLR5 and TLR8 stimulation, THP1-XBlue™ cells activate transcription factors and subsequently the secretion of SEAP which is easily detectable when using QUANTI-Blue™, a medium that turns purple/blue in the presence of SEAP. The results were analyzed using a microplate reader (Varioscan Lux, Thermo Scientific).

### 2.11. Galleria Mellonella Larvae Infection Model

The virulence of PAO1 mutants resistant to PA5oct phage was tested in vivo in *G. mellonella* infection model, previously described by Cullen et al. [34]. The wax moth larvae were sorted by size and weight and acclimated for one week at 15 °C. The larvae were infected by the injection into the hindmost proleg, of 10 µL of bacterial suspension (10^3^ cfu/mL) giving 10 cells per larvae. Next, the larvae were incubated at 37 °C for 72 h, and their viability was checked after 8, 18, 24, 48 and 72 hours post-injection. The two types of control were made: the negative (larvae injected with 10 µL of sterile PBS buffer) and positive (larvae infected with a wild PAO1 strain). The graphical presentation of the survival curves was prepared using GraphPad Prism 6.0 (GraphPad Software Inc., La Jolla, CA, USA). The log-rank Mantel-Cox test was used for statistical analysis (*P*-values < 0.05 were regarded as significant).

### 2.12. PA5oct Phage DNA Detection in Bacterial Clones

The presence of PA5oct genome within the cells of PAO1 resistant mutants was evaluated using a standard polymerase chain reaction (PCR). The DNA was isolated using PureLink Genomic DNA mini kit (Invitrogen, Thermo Scientific). On the basis of the full sequence of PA5oct genome, primers flanking a fragment of the structural gene (major head subunit precursor) were designed (F: 5’-GATACATACCCTACGTGTTCGTTATG-3’ and R: 5’-GCACCGTTACCCAGCGAGTTAG). The PCR was carried out under the optimized conditions: initialization (95 °C/5 min), 30 cycles of denaturation (95 °C/30 s), annealing (56.4 °C/1 min) and elongation (72 °C/1 min 10 s) followed by final elongation (72 °C/10 min). The resulting 872 bp reaction product was visualized by standard agarose gel electrophoresis (1% agarose, 1× TBE buffer, 95 V/cm/45 min). The positive control was a purified PA5oct phage preparation and the negative control was a PAO1 strain (Figure S4).

### 2.13. Real-Time PCR Quantification of Bacterial versus Phage DNA Copies Increment over Time

A bacterial suspension with an optical density of 0.5 McF was prepared from a 24-hour TSA plate culture using a densitometer (Densimat, Biomerieux, France) and serially diluted in PBS buffer. Suspensions containing approx. 10^6^ cfu/mL was used for the preparation of TSB bacterial culture (starting 10^5^ cfu/mL). The cultures were incubated for 4 h at 37 °C.

The semi-quantitative real-time PCR reactions were carried out directly on bacterial culture without prior DNA isolation. To track changes in the number of bacterial and phage genome copies over time, 2 µL of the culture from T0 (immediately after the culture was established) and T4 (after 4 hours of incubation at 37 °C) were used as templates for amplification. In each experiment, two parallel reactions (in three replicates) were carried out for each culture. Two sets of primers were designed. The first pair targeted a fragment of PA5oct phage major head subunit precursor gene (F: GATACATACCCTACGTGTTCGTTATG and R: CCAGAATATGCTTTTGCAATATCGAAC). The second pair targeted a fragment of the gene encoding 16S rRNA in *P. aeruginosa* (F: GCGCAACCCTTGTCCTTAGTT and R: TGTCACCGGCAGTCTCCTTAG). Control was composed of wild-type PAO1 culture. RT-PCR was performed using QuantStudio3 system (Applied Biosystems, USA) in accordance with the manufacturer’s instructions, with the reaction conditions: 96 °C for 4 min., followed by 40 cycles at 96 °C for 15 s and 60 °C for 45 s.

The results of RT-PCR experiments were analyzed by QuantStudio3 software (supplied by manufacturer) and presented as the ΔCT_bacterial_/ΔCT_phage_ ratio (CT—cycle threshold). Assuming the homogeneity of the population within each tested bacterial mutant, the results were interpreted as follows: ΔCT_bacterial_/ΔCT_phage_ < 0.8 means an increase in the number of phage DNA copies in relation to bacteria DNA as a result of phage propagation during 4-hour incubation as a result of lytic cycle; 0.8–1.3 means the equilibrium of phage and bacterial DNA copies as a result of the proliferation of phages along with bacteria; >1.3 means the predominance of bacterial DNA over phage DNA copies as a result of bacterial cell division rate over phage propagation rate suggesting phage-free cells separation within the population.

## 3. Results

### 3.1. The Host Range of Phage PA5oct Suggests an Increased Activity against Clinical CF Isolates

The lytic activity of PA5oct was examined on two independent *P. aeruginosa* panels. First, on the COST international reference panel of 43 clinical *P. aeruginosa* [34]. Within this collection, phage PA5oct infects 24% of the isolates, which is slightly lower compared to representatives of the *Luz7virus* LUZ7 (42%), *Phikmvvirus* group (LUZ19 44%), *Pbunavirus* group (LBL3 40%, KT28 28%, KTN6 42%) and *Phikzvirus* group (phiKZ 47%, KTN4 33%). A second collection of 47 cystic fibrosis isolates obtained from the Leuven University hospital, Leuven, Belgium showed different results (Tables S1 and S2). The activity on these isolates from long term chronic infections was much broader, with PA5oct showing productive infection on 40% of the collection, whereas phages such as phiKZ and LUZ19 have a narrower host-range (20% and 21% respectively). The COST international reference panel also contains 25 CF isolates but PA5oct phage only lyses six of them. No correlation could be observed in terms of phage activity versus CF early/late type of *P. aeruginosa* isolate. These results indicate the antibacterial potential of PA5oct against clinical *P. aeruginosa* strains isolated from different kinds of infections (burn wounds, CF-patients pneumonia, nosocomial pneumonia, and urinary tract infection).

Propagation experiments on a panel of specific PAO1 cell wall knock-outs reveals that PA5oct requires the presence of LPS and at least a second host cell surface receptor, like the Type IV pili (Table 1). The flagella mutant ΔfliC does not provide conclusive results concerning the susceptibility to phage infection.

### 3.2. Phage PA5oct Infection Causes Significant Reduction of Planktonic/Cell Line-Associated Bacteria in an Airway Surface Liquid Infection Model

The in vitro antibacterial activity of PA5oct against *P. aeruginosa* was assessed using the ASL model on normal NuLi-1 and cystic fibrosis CuFi-1 bronchial epithelium cell lines [20,28]. These two cell lines mimic the natural environment of lung in healthy and cystic fibrosis patients respectively. Three different, well-characterized *P. aeruginosa* strains were applied, including the model PAO1, a burn-infection strain nonCF0038, and a small colony variant CF708 that was isolated from the late stage of CF infection [35]. Epithelial cells viability controls were established as well, and no toxicity was observed for phage and bacterial samples.

**Figure 1:**
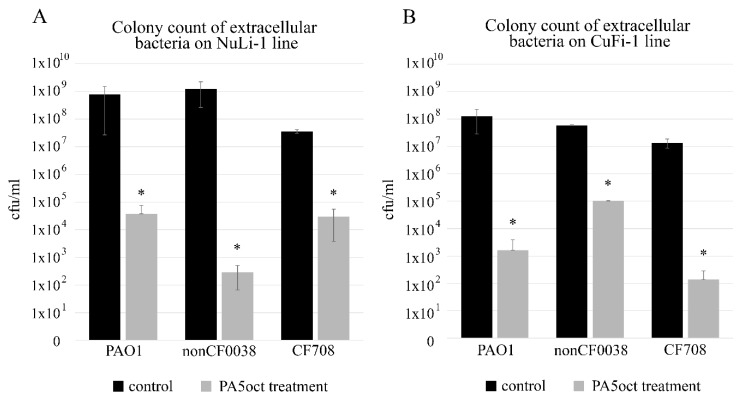
**Phage PA5oct treatment of *P. aeruginosa* infecting NuLi-1 (A) and CuFi-1 epithelial cells (B)**. Colony count of bacteria collected from apical wash. The gray bars represent bacteria titers after 1.5 h of incubation with PA5oct phage. The black bars represent controls without phage treatment. The error bars indicate the standard deviation. The results are presented as the means ± SD. Statistical analysis was made using the ANOVA test (denoted *p*-values < 0.05). (*) the instance with *p*-values < 0.05.

Quantification of bacterial cells was done by plating of serially diluted apical washes of cell cultures. After infection of both epithelial cell lines for 3 h, the colony count showed that all *P. aeruginosa* strains efficiently propagated in both ASL setups (10^7^–10^9^ cfu/mL). Phage treatment significantly (*p* < 0.05) reduced the CFU counts for normal NuLi-1, where 4.5 log, 6.5 log and 3 log decreases were observed for PAO1, nonCF0038 and CF708, respectively (Figure 1A). The phage application for CuFi-1 epithelia infection was also very effective (*p* < 0.05) giving 5 log, 2.5 log and 5 log reductions in CFU of PAO1, nonCF0038 and CF708, respectively (Figure 1B).

### 3.3. Real Time Measurement of P. aeruginosa Biofilm Diffusion Properties

**Figure 2:**
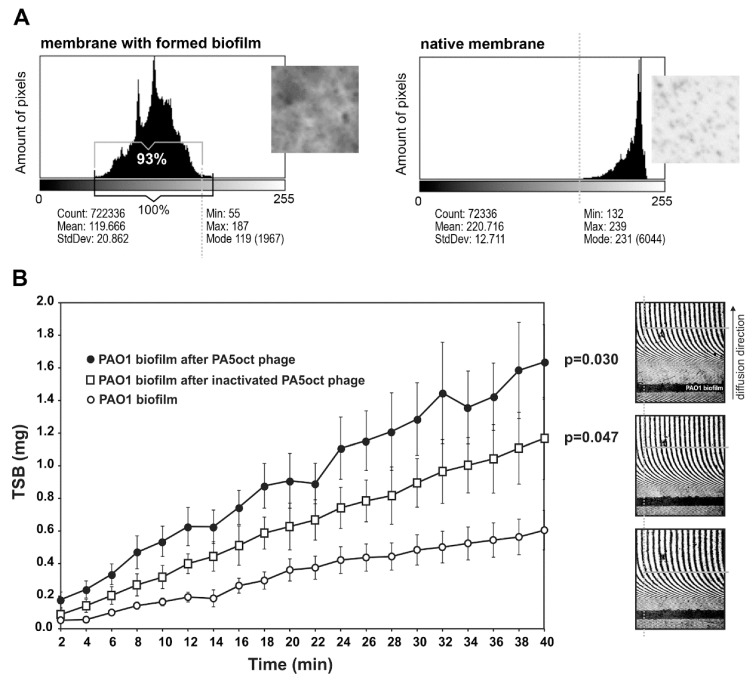
Real time measurement of biofilm permeability, after 4 h of phage treatment. (**A**) Membrane coverage analysis by crystal violet (CV) staining and ImageJ imaging software. Native membrane was used as a control. (**B**) Laser interferometry analysis of trypticase-soy broth (TSB) medium diffusion through PAO1 biofilm treated with PA5oct phage. Untreated biofilm was used as a control. Error bars denote SD. The results displayed are the mean of three independent experiments. Statistical analysis was made by the ANOVA test to compare data of treated biofilm versus control native biofilm at 40 min time point. The examples of interferograms (40 min) for PAO1 biofilm treated with active and inactivated PA5oct phages as well as control (from the top of a right-hand panel).

The direct effect of PA5oct phage on the mature (72 h) biofilm *P. aeruginosa* PAO1 (overspread on hydrophilic Nephrophane membrane scaffolding) was investigated using laser interferometry, a standardized approach used for diffusion properties measurement, which allows better comparison to other experiments on how the biofilm biomass is degraded [20,23]. First, the membrane was examined in terms of the biofilm coverage. Photos of membranes stained with crystal violet (CV) were collected and converted into grey-scale digital images (1 denotes black and 256 denotes white) (Figure 2A). Using the ImageJ computer imaging software [36] the degree of membrane coverage by biofilm was estimated at around 93%. Second, the biofilm was treated with active or inactive phages for 4 hours and then washed out to remove phage suspensions. Next, the diffusion event was measured by interferometry for 40 min in real-time (Figure 2B). An increase of TSB diffusion rate through the biofilm layer correlates with the structural degradation of the biofilm/matrix. The diffusion rate of medium transported through the intact biofilm-covered membrane after 40 min (0.605 mg) was significantly lower than for biofilm (*p* < 0.05) after active and inactivated phage treatment, reaching 1.64 mg and 1.17 mg, respectively. These experiments indicated that phage PA5oct as infecting virions as well as inactivated virions was able to reduce the density of *Pseudomonas* biofilm after 4 h-treatment. The increase in diffusion obtained after the application of inactivated particles may be explained by an enzymatic activity of virion-associated proteins responsible for biofilm matrix degradation.

**Figure 3:**
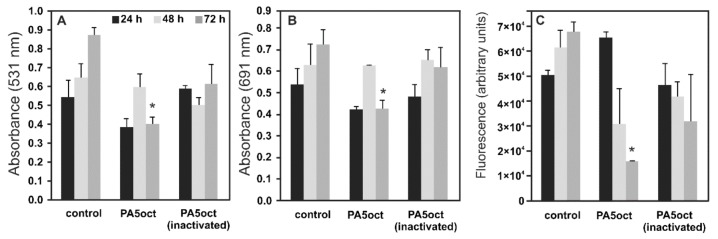
The anti-biofilm effect of PA5oct phage treatment (4 h) on 24, 48 and 72 h PAO1 biofilm formed on Nephrophane membrane. The biomass evaluation by CV staining (**A**); the level of pyocyanin in growth medium (**B**); the fluorescence of pyoverdin in growth medium (**C**). Untreated biofilm was used as control. The results are presented as the means ± SD. Statistical analysis was made by the ANOVA test (denoted *p*-values < 0.05). (*) the instance with *p*-values < 0.05.

To establish the antimicrobial activity of phage PA5oct against sessile bacteria, three complementary assays were performed: the biomass CV staining and the measurement of pyocyanin and pyoverdin/pyochelin secretion (Figure 3). Experiments were performed on a Nephrophane membrane with overgrown PAO1 biofilm, at various time points (24, 48 and 72 h). The CV staining of biofilm biomass showed a significant effect of the active PA5oct against mature biofilm (72 h). Moreover, the analysis of pyocyanin and pyoverdin/pyochelin secretion indicated that active phages significantly decreased the level of these compounds in the biofilms tested (72 h), whereas there was no such clear effect in the case of UV-inactivated phages. A positive dependence between biofilm formation, pyocyanin and pyoverdin/pyochelin levels was observed in the supernatant, indicating that a reduced level of *Pseudomonas*-specific compounds was related to phage activity. In summary, we show that phage PA5oct primarily affects mature biofilm, reducing its biomass as well as inhibiting the production of selected virulence factors.

### 3.4. Phage PA5oct Impact on PAO1 Biofilm-Living Population after Infection

An important aspect of this study was to evaluate the occurrence of PA5oct phage resistance within infected biofilm population of *P. aeruginosa*. For this purpose, thirty colonies were randomly selected from 24, 48 and 72-hours biofilm after phage treatment and checked for phage susceptibility. Simultaneously, thirty control clones from an untreated biofilm were also sampled. A third of phage-exposed clones (10/30) and all control isolates still remained susceptible to PA5oct infection, whereas 20 clones had become resistant. Such a high percentage of clones still sensitive to phage is probably due to the presence of persister cells in the bacterial population. This type of dormant cell shows a low metabolism and protein expression, which reduces the sensitivity to both bacteriophages and antibacterial agents. The biological function of persister cells is to restore a population that has been depleted for some reason. It is worth mentioning that from an ecological and evolutionary point of view, the extermination of host by predator is unlikely thus the complete eradication of the bacterial population is not observed for phage propagation [37].In the next step, isolates were examined for cross-resistance to other phages lytic to PAO1 wild type strain (Table S1), recognizing different receptors: Type IV pili-dependent (phiKZ, KTN4, LUZ19) and LPS-dependent (KT28, KTN6, LUZ7, LBL3). Control isolates and PA5oct-sensitive clones taken from the biofilm after phage exposure retain the same phage typing pattern as PAO1 wild type. In total, six different phage typing patterns (PA5oct-sensitive and resistant types 1–5) could be distinguished (Table 2).

In most of the cases, the cross-resistance patterns were related to *Pbunavirus* phages (*Myoviridae*), which primarily target the LPS structure (type 5). However, some PA5oct-resistant clones were resistant to LBL3 phage (*Pbunavirus*) and Type IV pili-dependent giant phages (phiKZ and KTN4). These clones were still susceptible to phages KT28 and KTN6. Three mutants exhibiting resistance to KTN4 phage remained susceptible to its (genomically) closely related phiKZ counterparts (>99% genome-wide DNA homology) [20]. These results indicate that PA5oct requires two different receptors for an effective infection. Interestingly, no cross-resistance was observed for podoviruses LUZ7 and LUZ19, despite their dependence on the same bacterial surface macromolecules for infection (LPS and Type IV pili, respectively).

### 3.5. Emerging Phage PA5oct-Resistant Clones Showed a Reduced Virulence

To evaluate the correlation between phage receptor modification in emerging phage-resistant populations and the principal virulence factors of these clones, we compared LPS patterns, twitching motility (Type IV pili dependent), growth rate, and in vivo pathogenicity in *Galleria mellonella* model (Table 3).

The analysis of LPS extracted from PA5oct phage-resistant types (type 1–5) shows no major changes compared to both—PA5oct-sensitive mutants and biofilm-derived controls (Figure S1). No truncation of the O-specific LPS chain was observed in any case. In the context of these results, cross-resistance to LPS-dependent (KT28, KTN6, LBL3, LUZ7) bacteriophages is an interesting observation. The evaluation of Type IV pili-dependent twitching motility revealed a significant reduction among all the phage-exposed bacteria tested (including PA5oct-sensitive), compared to control isolates. Changes in Type IV pili expression may be an explanation for the cross-resistance to pili-dependent phages (KTN4, phiKZ) occurring in 5 out of 20 PA5oct-resistant isolates (Table 3).

The evaluation of growth rate revealed that 8 out of 10 selected PA5oct-sensitive clones remained similar to the growth of parental PAO1 and to 30 biofilm-derived controls (OD_590_ > 1.5 after 24 h) (Table 3). Among PA5oct-resistant clones 16 out of 20 showed a steep decrease of the growth rate (OD_590_ < 1.0 after 24 h) (Table 3, Figure S2).

**Figure 4:**
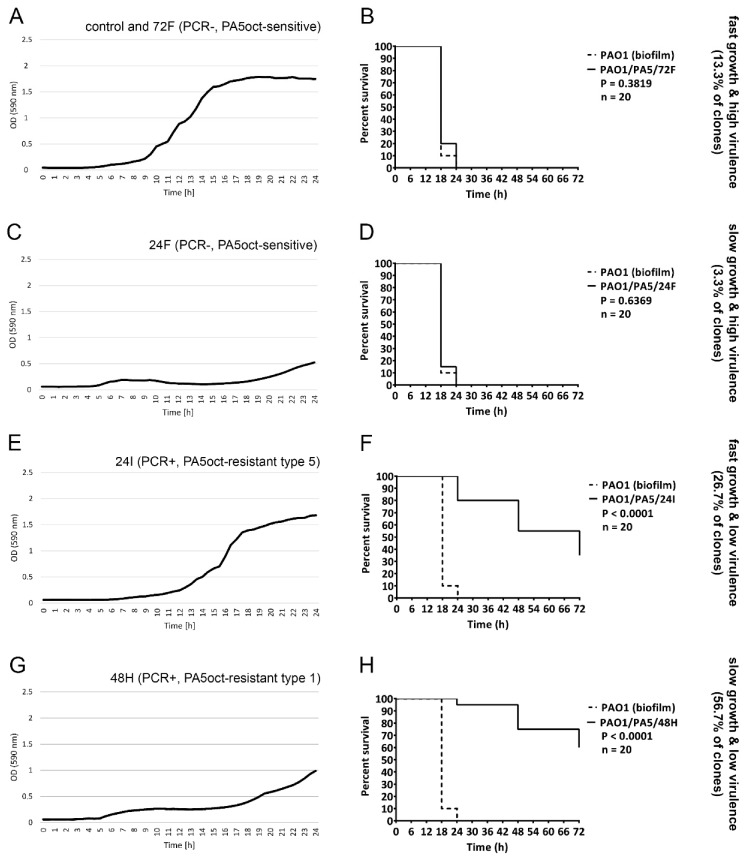
Phenotypic patterns in PAO1 clones treated with PA5oct. Growth curves (**A**,**C**,**E**,**G**) of selected bacterial clones combined with a survival of infected *G. mellonella* (**B**,**D**,**F**,**H**) for PA5oct-sensitive and PA5oct-resistant clones. Fast-growing with a high virulence (control and 72F; (**A**)/(**B**)); slow-growing with a high virulence (24F; (**C**)/(**D**)); fast-growing with a low virulence (24I; (**E**)/(**F**)), and slow-growing with a low virulence (48H; (**G**)/(**H**)). Data for all clones are presented in Supplementary Figure S2.

The impact on the biological features listed above hints towards a modification of the bacterial virulence, which could prove to be a key strategic advantage for the use of PA5oct in therapeutic settings. To validate this observation, we examined the virulence in vivo, using the *G. mellonella* infection model (Table 3, Figure 4, data for remaining clones in Supplementary Figure S2). Using equal doses of bacteria (10 µL of 10^3^ cfu/mL) 25 out of 30 tested clones showed a significant decrease in virulence (*P* < 0.05), including 5 PA5oct-sensitive clones. We were finally able to distinguish four distinct phenotypic patterns in PAO1 clones: fast-growing with a high virulence (13.3% of clones, Figure 4A,B); slow-growing with a high virulence (3.3% of clones, Figure 4C,D); fast-growing with a low virulence (26.7% of clones, Figure 4E,F), and slow-growing with a low virulence (56.7% of clones, Figure 4G,H). The relationship between growth rate and virulence of all phage-treated bacterial mutants are summarized in Table 3 and Figure S2.

### 3.6. The Reduced Virulence in PA5oct-Resistant Clones Is Correlated with the Persistence of Phage in Bacterial Population

The analysis of 30 phage-treated clones indicates that the reduced virulence has emerged as a consequence of PA5oct infection of the PAO1 population. Moreover, slow-growing isolates may suggest a phage propagation event in the bacterial culture. Therefore, a PCR assay targeting the unique gene encoding the major head subunit precursor in PA5oct was implemented to confirm the presence of phage DNA within bacterial population. All PA5oct-resistant isolates and one still sensitive (72C) were PCR-positives (Table 3) and all those clones were less virulent compared to wild type PAO1. Moreover, half of the clones that were still sensitive to phage infection became less virulent as well. A high prevalence of phage maintenance within resistant bacterial cells might suggest prolonged phage propagation or pseudolysogeny/carrier state event.

To evaluate the propagation dynamics of PA5oct phage present in resistant mutant populations, we used semi-quantitative real-time PCR reactions as an indirect method to calculate the difference in the number of bacterial and phage DNA copies at the beginning and the end of 4-hour incubation. The results were presented as ΔCT_bacterial_/ΔCT_phage_ ratio (Table 3). Assuming homogeneity of isolated PAO1 mutants population, the calculated ΔCT ratios revealed that in 19 out of 20 PA5oct phage-resistant mutant cultures the PA5oct genome replicates along with the bacterial genome (state of equilibrium, ΔCT_bacterial_/ΔCT_phage_ = 0.8–1.3). In the case of ΔCT_bacterial_/ΔCT_phage_ > 1.3 (72C and 24A clones), the results indicate a faster rate of replication of bacterial genome. The predominance of bacterial DNA over phage DNA copies suggests phage-free cells separation within the population.

In conclusion, no case has been reported where the genome of PA5oct phage would multiply faster than the genome of the bacterium suggesting no or minimal level of phage propagation in the lytic cycle within phage-resistant clones.

### 3.7. The Presence of PA5oct in PAO1 Population Induce Pro-Inflammatory Response in Monocytes

Given the results listed above, we were interested to investigate the possible influence of PA5oct infection on pro-inflammatory features of *Pseudomonas* population, which would further support the antimicrobial activity obtained in the ASL and larvae models. Toll-like Receptors (TLRs) present on phagocytic cells serve as Pattern Recognition Receptors (PRRs) and play a crucial role in the proper functioning of the innate immune system. Therefore, we selected 10 PA5oct-resistant clones and determined the TLR stimulation profile of THP1-XBlue™ monocytes line when treated with bacteria culture filtrates. The results correlated perfectly to our in vivo data, in which the same mutants exhibiting a low virulence and PCR-positive, gained pro-inflammatory features, strongly stimulated the monocyte culture, relative to PAO1 isolate controls (Figure S3).

## 4. Discussion

The main aim of this study was to evaluate the influence of PA5oct bacteriophage on the population of *P. aeruginosa* PAO1 strain, both in the context of the whole population and individual mutants isolated after phage infection. Phenotypic changes appearing in mutant strains and their significance for virulence were particularly analyzed. Based on phage typing of knock-out strains and cross-resistance results, it was found that phage PA5oct recognizes both LPS and type IV fimbriae, and other receptors cannot be excluded. This versatility can generally explain the broad spectrum of lytic activity of PA5oct phage and jumbo phages in general. In our case, the distinct host range between both strain panels is noteworthy. In the reference panel of *P. aeruginosa* (BCCM/LMG), containing 43 strains (25 isolated from patients with cystic fibrosis) [38], only 24% of isolates showed the susceptibility to PA5oct phage (6 from CF patients) [34]. Concurrently, phage PA5oct is able to infect 40% of isolates from our standard clinical strain panel. This is probably due to the specific phenotype of long-term CF-related strains, including reduced virulence patterns compared to environmental strains (modified LPS structure, lower expression of Type IV pili and flagellum and a decrease of production intensity of alginate, pyocyanin, pyoverdine, and elastase) [39,40,41].

We used the ASL model to mimic natural conditions of *Pseudomonas* epithelium infection in vitro. The experiments confirmed that PA5oct can efficiently access and infect planktonic, mucus embedded and cell-adhered bacteria, but remained dependent on the strain used, as previously observed for giant *Pseudomonas* KTN4 phage [20,42]. According to a study by Worlitzsch et al. [42] using **CuFi-1 epithelial** cell line, *P. aeruginosa* does not interact with the CF epithelium directly, but rather gets embedded in mucus plugs formed in the airways. Therefore, in CF patients, the potential use of phages in therapy should be preceded by studies on the ability of particular phage to penetrate the mucus plugs.

*P. aeruginosa* strains entering the human respiratory tract usually express the entire arsenal of virulence factors including flagellum, LPS, Type IV fimbriae or alginate [43]. It enables bacteria to successfully adhere to and invade respiratory epithelium. Simultaneously, the expression of bacterial surface structures makes bacteria more susceptible to potential phage infection (phages usually target LPS and Type IV fimbriae). Under natural conditions, bacterial infection stimulate the pro-inflammatory response of immune and epithelial cells, which results in an increased production of antibacterial peptides, cytokines and recruitment of phagocytic cells [44,45,46,47,48]. In the experimental case of epithelial NuLi-1 and CuFi-1 cell lines [28], a significant decrease in bacterial cell counts demonstrated in ASL studies may be linked to the synergistic action of PA5oct phage and antibacterial peptides produced by the epithelial cell lines tested. Moreover, according to THP1-XBlue™ cell line experiments, the release of pro-inflammatory compounds from *Pseudomonas* populations infected with PA5oct phage resulted in increased stimulation in monocytic cells enhancing the clearance mechanisms of innate immune system. This further supports our results in both ASL and in vivo model.

From the perspective of sessile cells, the infection by phages is limited due to the presence of biofilm matrix, the modification of bacterial surface receptors and reduced metabolic activity [1,49]. Apart from being able to diffuse in a dense airway mucus, PA5oct could also get an access to bacterial cells hidden in a mature 72-hour biofilm. This was verified by our two complementary assays (permeability assay and Nephrophane membrane biofilm assay). That observation is in contradiction with the general view that phages have the greatest activity against immature biofilms composed mostly of metabolically active cells [50,51]. In the case of PA5oct, biofilm biomass reduction occurred only as a result of phage-mediated cell lysis, whereas biofilm matrix was degraded by both active and inactivated phages (shown by a quantifiable increase of TSB medium diffusion trough the Nephrophane membrane).

The plaques produced by PA5oct phage are very small with no halo zones. Nonetheless, the *in silico* analysis of PA5oct virion-associated proteins using Phyre2, UniProt BLAST, BLASTP, HMMER, and SwissModel (EsPASy) software identifying potential enzymes able to degrade bacterial exopolysaccharide matrix. Such a result may suggest that PA5oct phage destroys the biofilm not only by unsealing its structure after tightly packed cells lysis, but it might also use exopolysaccharide depolymerases (potential candidates gp162 and gp205), which are probably responsible for matrix disruption [52,53]. The gp162 was the first hit with 100.0% confidence in the model (N-term: tail connector protein/centre: transferase/C-term: lyase (351–451 aa) forming putative tail sheath stabilizer or envelope glycoprotein. The second hit was gp205 a putative hydrolase alpha-n-acetylglucosaminidase (66.5% confidence in the model) catalyzing the hydrolysis of glycosidic bonds in complex sugars. In the case of halo-exhibiting phages, mostly Podoviridae, the zone occurs as an effect of depolymerase diffusion or phage diffusion in the agar. In the case of giant phages with virion-associated enzymes the diffusion in the agar is strongly limited by the size of phage capsid, thus the halo zone might not be observed [54].

The introduction of PA5oct phage into the biofilm population of *P. aeruginosa* triggered the emergence of clones resistant to this virus. The PA5oct-resistant isolates subjected to typing with a panel of phages recognising different receptors, showed that the phenotypic changes under the influence of PA5oct pressure went beyond the modulation of a single receptor. PA5oct-resistant isolates showed a cross-resistance to phages belonging to different taxonomic units, and the typing patterns were diverse, suggesting genomic rearrangements and multiple mutations in the bacterial genome which impact multiple receptors [31,55,56,57]. These changes in the genome of the host are currently under investigation by our team.

The formation of phage-resistant bacterial variants is often considered as a limitation to the implementation of phage therapy. Although phage-related phenotypic changes in bacteria decrease the efficacy of phage therapy as a standalone therapy, it turns out that in practice the occurring phenotypic modifications usually lead to a diminished bacterial virulence [58,59]. Consequently, the *Pseudomonas* population that survived the phage treatment/infection became sensitive to the immune system that effectively removes the pathogenic agent. Our findings were confirmed in the *G. mellonella* infection model, but also reported by others in murine model [7]. Nevertheless, we can deduce that a PA5oct phage infection leads to the emergence of less-virulent phage-resistant clones, but this phenomenon is no longer so obvious for clones that remain sensitive to phage (50% of PA5oct-sensitive clones was still highly virulent).

An often neglected phenomenon is the occurrence of pseudolysogeny/carrier stage, generally regarded as a temporary stage of phage particle dormancy. However, it appears that the presence of a phage episome inside the host cell influences its phenotype and contributes the cross-resistance to other bacteriophages as well [60,61,62]. The jumbo phages are known to easily undergo episome formation as was previously reported for phiKZ phage [10]. In this study, we indicated the presence of PA5oct DNA in isolated resistant-clones and linked it to modified virulence of the PAO1 population carrying the phage DNA. A significant survival improvement was seen in larvae infected with those isolates. These strains became significantly less pathogenic in vivo compared to wild-type PAO1 and to PCR-negative phage-resistant mutants. This effect could potentially be directly or indirectly induced by the expression of specific PA5oct genes. Taking into account that the prevalence of PCR-positive clones was relatively high (21/30 strains) we may conclude that this specific jumbo phage PA5oct efficiently eradicates sensitive *P. aeruginosa* cells both planktonic and sessile, while at the same time selects for a primarily non-virulent pseudolysogenic/carrier resistant population. The determination of phage or phage genome location within an infected bacterial population is an extremely complex task. Without a direct tagging of phage DNA [63], it is not possible to clearly determine whether the product multiplied by the PCR reaction originates from the DNA of a prophage, an episome or a free phage virion. Proof of phage maintenance within the cells could be only done at a single cell level. The results of experiments using RT-PCR presented in this paper indirectly determine whether the increase in the number of phage genome copies results from an active propagation (lytic cycle) or passive replication of viral genome in episomal/carrier form.

The jumbo phages are reported to perform generalized transduction, which drives bacterial evolution and can cause the rapid spread of dangerous antibiotic resistance or virulence genes. On the other hand, for therapeutic purposes, phages have to be propagated only on the non-virulent bacterial strains. In addition, it is possible to isolate phage mutants with reduced frequency of transduction [10]. The obstacle presented by generalised transduction cannot eliminate jumbo phages from the discussion about phage therapy. It is worth remembering that even in the worst scenario, where the bacteriophage capsid is packed with bacterial genes, most often the possibility of transmission is low due to the very rapid clearance of phages by mononuclear phagocytic system [64].

Owing to its unusual behavior when compared to other known virulent phages, PA5oct proved to be an interesting subject for research in the ecological and evolutionary context. Its anti-biofilm activity and the influence on bacterial virulence factors expression exert a strong selective pressure within the bacterial population. This phenomenon, combined with the long-term persistence of PA5oct in the bacterial population (pseudolysogeny/carrier stage) and the generation of cross-resistance to unrelated species of viruses, is an important item for further research that can contribute to our understanding of co-evolution of bacteria and their phages. The pro-inflammatory activity of bacterial clones infected by phages causing an increased susceptibility to the innate immune system is another interesting aspect that is currently being investigated.

## Figures and Tables

**Table 1:** Phage receptor identification on *P. aeruginosa* PAO1 mutants.

PAO1 Isolates	Phenotype	PA5oct Activity
ATCC 15692 (PAO1)	Wild type	+
Δrmd (A−, B+) *	Deficiency in D-rhamnose biosynthesis; lack of A-band LPS	+
ΔwaaL (A−, B−) *	Lack of WaaL ligating O-polymer to core-lipid A; LPS is devoid of A-band and B-band, semirough (SR-LPS, or core-plus-one O-antigen)	−/+
ΔwbpL (A−, B−) *	Lack of glucosyltransferase WbpL essential for initiation of both A-band and B-band synthesis	−
ΔfliC ΔalgC ΔpilA **	Lack of flagella; lack of AlgC required for A-band, core oligosaccharide, and alginate biosynthesis; lack of Type IV pili	−
ΔfliC ΔpilA **	Lack of flagella; lack of Type IV pili	−
ΔfliC WTpilA **	Lack of flagella	−/+

(+)—clear plaque; (−)—lack of lytic activity; (+/−)—opaque plaque; * Laboratory of Foodborne Zoonoses, Guelph, Canada, Andrew M. Kropinski; ** Technical University Hamburg, Germany, Max Schöbert.

**Table 2:** Phage typing of PA5oct clones obtained during biofilm treatment.

Bacterial Clones	Susceptibility to Phage Infection								Number of Isolates
	LPS/Pili	LPS-Dependent				Pili-Dependent			
	PA5oct	LBL3	KT28	KTN6	LUZ7	KTN4	phiKZ	LUZ19	
Control planktonic PAO1	+	+	+	+	+	+	+	+	1
Control biofilm PAO1	+	+	+	+	+	+	+	+	30
PA5oct sensitive	+	+	+	+	+	+	+	+	10
PA5oct resistant type 1	−	−	+	+	+	−	+	+	1
PA5oct resistant type 2	−	−	+	+	+	−	−	+	2
PA5oct resistant type 3	−	+	−	−	+	−	+	+	2
PA5oct resistant type 4	−	+	−	−	+	+	+	+	5
PA5oct resistant type 5	−	−	−	−	+	+	+	+	10

(+) sensitive to phage infection; (−) resistant to phage infection.

**Table 3:** The virulence features of *P. aeruginosa* clones after PA5oct treatment.

Bacterial Clones	Name	LPS Pattern	Growth Rate [OD_590_/24 h]	Larvae Survival Rate [%] (18/24/48/72 h)	Twitching Motility [mm]	Phage DNA Presence (PCR)	ΔCT PAO1/ΔCT PA5oct Ratio
control planktonic	PAO1	S	>1.5	10/0/0/0	24.2 ± 1.3	−	−
control biofilm	K72A	S	>1.5	30/0/0/0	23 ± 2.0	−	−
control biofilm	K72B	S	>1.5	30/0/0/0	22.9 ± 1.9	−	−
control biofilm	K72C	S	>1.5	10/0/0/0	22.8 ± 2.3	−	−
control biofilm	K72D	S	>1.5	5/0/0/0	22.7 ±1.5	−	−
control biofilm	K72E	S	>1.5	15/0/0/0	23.5 ± 1.3	−	−
control biofilm	K72F	S	>1.5	0/0/0/0	23.8 ± 1.2	−	−
control biofilm	K72G	S	>1.5	5/0/0/0	23.7 ± 1.6	−	−
control biofilm	K72H	S	>1.5	35/0/0/0	23.3 ± 2.1	−	−
control biofilm	K72I	S	>1.5	15/0/0/0	23.8 ± 1.1	−	−
control biofilm	K72J	S	>1.5	25/0/0/0	23.2 ± 1.5	−	−
PA5oct sensitive	24F	S	<1.0	15/0/0/0	18.2 ± 0.9 *	−	−
PA5oct sensitive	48B	S	>1.5	20/10/0/0	18.5 ± 1.0 *	−	−
PA5oct sensitive	48C	S	>1.5	80/35/0/0 **	19.3 ± 1.3 *	−	−
PA5oct sensitive	48F	S	>1.5	30/0/0/0	15.7 ± 0.8 *	−	−
PA5oct sensitive	48G	S	>1.5	80/40/15/0 **	19 ± 0.8 *	−	−
PA5oct sensitive	72B	S	>1.5	30/25/10/0 **	18.4 ± 1.3 *	−	−
PA5oct sensitive	72C	S	<1.0	80/55/10/0 **	15.5 ± 1.3 *	+	1.7
PA5oct sensitive	72E	S	>1.5	55/25/0/0 **	14.6 ± 0.7 *	−	−
PA5oct sensitive	72F	S	>1.5	20/0/0/0	18.3 ± 0.8 *	−	−
PA5oct sensitive	72G	S	>1.5	30/15/0/0	18.5 ± 1.1 *	−	−
PA5oct resistant type 1	48H	S	<1.0	100/95/75/60 **	5.5 ± 1.3 *	+	0.8
PA5oct resistant type 2	24C	S	<1.0	75/60/55/35 **	15.4 ± 1.0 *	+	0.8
PA5oct resistant type 2	48A	S	<1.0	70/65/30/0 **	17.5 ± 1.8 *	+	1.0
PA5oct resistant type 3	48E	S	<1.0	100/45/0/0 **	19.1 ± 1.3 *	+	0.9
PA5oct resistant type 3	72H	S	<1.0	95/55/10/0 **	13.6 ± 1.6 *	+	0.8
PA5oct resistant type 4	24A	S	<1.0	50/15/15/15 **	16.7 ± 2.0 *	+	2.0
PA5oct resistant type 4	24D	S	>1.5	75/60/40/20 **	11 ± 2.3 *	+	1.0
PA5oct resistant type 4	48I	S	<1.0	85/60/20/0 **	13.2 ± 0.6 *	+	0.9
PA5oct resistant type 4	48J	S	<1.0	80/60/15/15 **	15.4 ± 1.2 *	+	1.1
PA5oct resistant type 4	72I	S	<1.0	55/50/5/0 **	14 ± 1.1 *	+	1.2
PA5oct resistant type 5	24B	S	<1.0	85/80/40/40 **	16.5 ± 1.6 *	+	1.0
PA5oct resistant type 5	24E	S	<1.0	75/70/55/30 **	15.4 ± 1.6 *	+	1.0
PA5oct resistant type 5	24G	S	<1.0	75/70/65/35 **	12.5 ± 2.1 *	+	1.0
PA5oct resistant type 5	24H	S	<1.0	95/75/45/35 **	12.2 ± 1.3 *	+	0.8
PA5oct resistant type 5	24I	S	>1.5	100/80/55/35 **	13.3 ± 0.8 *	+	1.0
PA5oct resistant type 5	24J	S	>1.5	85/70/50/30 **	14.5 ± 1.7 *	+	1.3
PA5oct resistant type 5	48D	S	<1.0	85/45/15/0 **	14.4 ± 0.7 *	+	0.8
PA5oct resistant type 5	72A	S	<1.0	95/70/0/0 **	12.5 ± 1.7 *	+	1.0
PA5oct resistant type 5	72D	S	>1.5	100/75/15/0 **	13.2 ± 0.9 *	+	0.9
PA5oct resistant type 5	72J	S	<1.0	95/80/10/0 **	22.8 ± 1.3 *	+	1.0

(S)—smooth LPS type; (*) significantly lower TM zone compared to control clones (One way ANNOVA, *P* < 0.05); (**) significantly higher *G. mellonella* larvae survival rate compared to infected by control clones (Log-rank (Mantel-Cox), *P* < 0.05; ΔCT PAO1/ΔCT PA5oct ratio: <0.8—an increase in the number of phage DNA copies in relation to bacteria DNA; 0.8–1.3—equilibrium of phage and bacterial DNA copies; >1.3—the predominance of bacterial DNA over phage DNA copies.

## Supplementary Materials

The following are available online at https://www.mdpi.com/1999-4915/11/12/1089/s1, Table S1. Main features of *Pseudomonas* phages used in the study. Table S2. Phage activity comparison of six virulent phages on *P. aeruginosa* strains from the University hospital of Leuven, Leuven, Belgium collection. Figure S1. LPS profiles of PAO1 clones isolated after controlled infection by PA5oct phage, analyzed in 14% polyacrylamide/tricine-SDS gels. Panels A, B, and C—isolates after phage treatment; Panel D—control isolates (untreated). Figure S2. Comparison of the growth rate, virulence (on *G. mellonella* model) and phage resistance pattern of 30 clones of *P. aeruginosa* PAO1 isolated as a result of controlled PA5oct phage infection. Figure S3. THP-1 X-blue monocyte response to PA5-phage-resistant clones post-culture medium stimulation. Figure S4. PCR analysis targeting the major head subunit precursor gene in selected PA5oct resistant clones. M-mass marker.
