# Supplementary material for: Pseudomonas aeruginosa PA5oct Jumbo Phage Impacts Planktonic and Biofilm Population and Reduces Its Host Virulence
Source: Viruses. 2019 Nov 23;11(12):1089. doi: 10.3390/v11121089 (PMC6950013; doi:10.3390/v11121089)
Supplement: Supplementary file 1 [file viruses-11-01089-s001.zip › olszak supplementary files/Table S1.docx]

**Table S1**. Main features of *Pseudomonas* phages used in the study

| **Name** | **Taxonomy** | **Recognized bacterial receptor** |
| --- | --- | --- |
| PA5oct | myovirus, giant** | LPS/IV type pili |
| 14/1 | Pbunavirus, myovirus* | LPS |
| phiKZ | Phikzvirus, myovirus, giant* | IV type pili |
| LUZ19 | Phikmvvirus, podovirus* | IV type pili |
| LKD16 | Phikmvvirus, podovirus* | IV type pili |
| KMV | Phikmvvirus, podovirus* | IV type pili |

*Laboratory of Gene Technology, KU Leuven, Leuven, Belgium

**Department of Pathogen Biology and Immunology, Institute of Genetics and Microbiology, University of Wroclaw, Wroclaw, Poland
