# Supplementary figures and images for: Pseudomonas aeruginosa PA5oct Jumbo Phage Impacts Planktonic and Biofilm Population and Reduces Its Host Virulence
Source: Viruses. 2019 Nov 23;11(12):1089. doi: 10.3390/v11121089 (PMC6950013; doi:10.3390/v11121089)

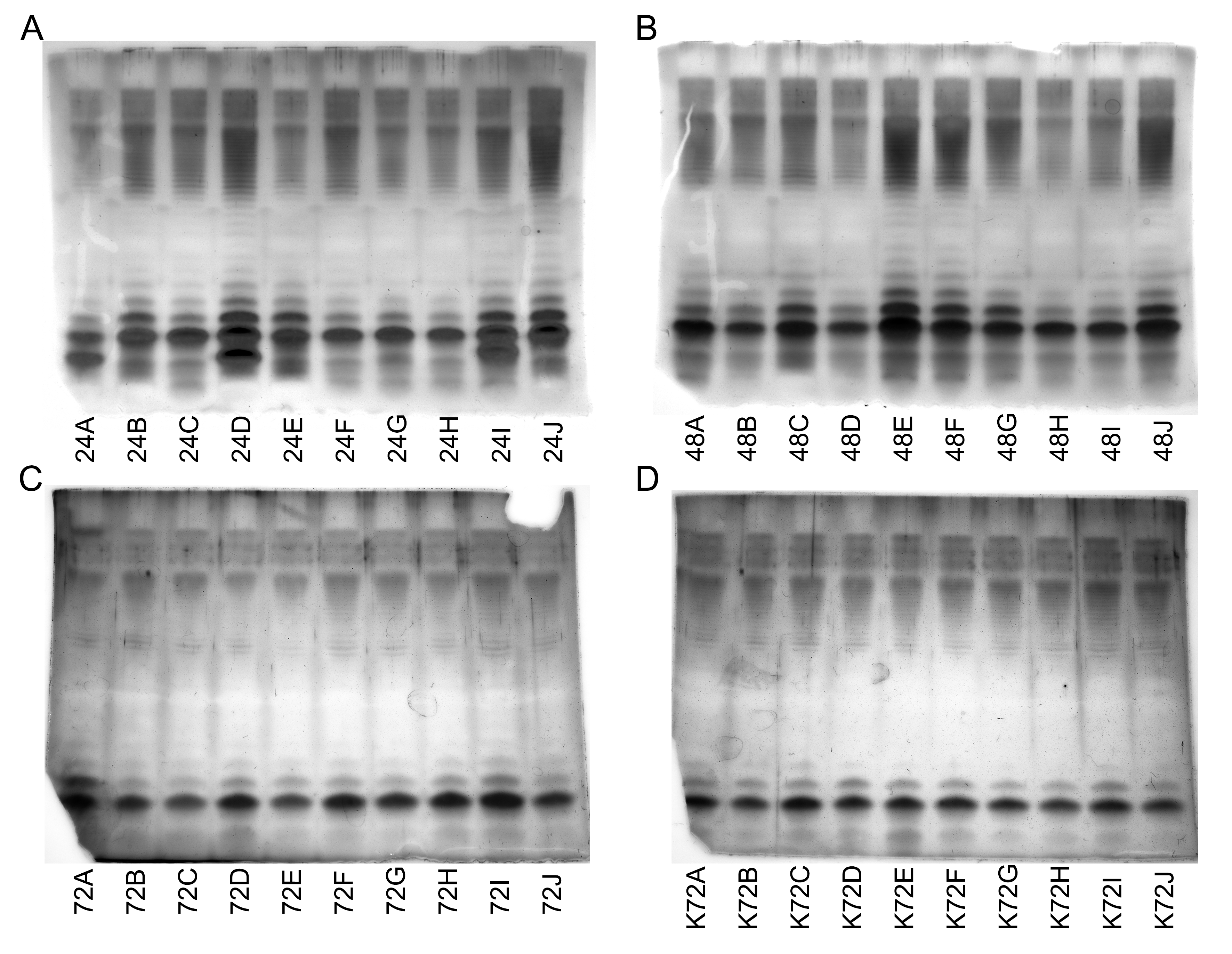

Supplement: Supplementary file 1 [file viruses-11-01089-s001.zip › olszak supplementary files/Figure S1.tif]

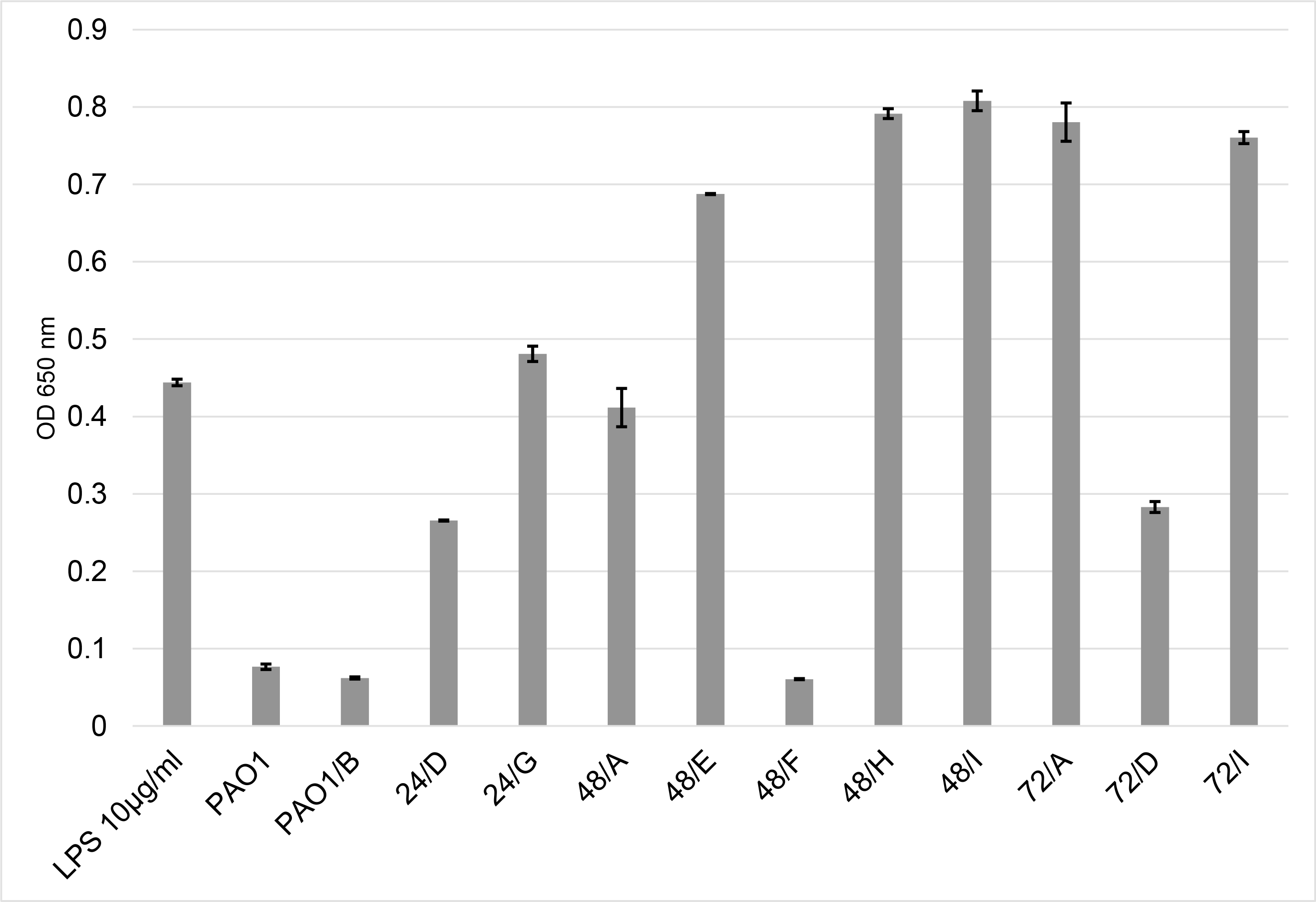

Supplement: Supplementary file 1 [file viruses-11-01089-s001.zip › olszak supplementary files/Figure S3.tif]
